# A 13-LOX participates in the biosynthesis of JAs and is related to the accumulation of baicalein and wogonin in *Scutellaria baicalensis*

**DOI:** 10.3389/fpls.2023.1204616

**Published:** 2023-07-13

**Authors:** Dali Geng, Rongyu Wang, Ya Zhang, Heng Lu, Hongjing Dong, Wei Liu, Lanping Guo, Xiao Wang

**Affiliations:** ^1^ Shandong Analysis and Test Center, Qilu University of Technology (Shandong Academy of Sciences), Jinan, China; ^2^ School of Pharmaceutical Sciences, Qilu University of Technology (Shandong Academy of Sciences), Jinan, China; ^3^ Institute of Traditional Chinese Medicine, Shandong Hongjitang Pharmaceutical Group Co., Ltd., Jinan, China; ^4^ State Key Laboratory Breeding Base of Dao-di Herbs, National Resource Center for Chinese Materia Medica, China Academy of Chinese Medical Sciences, Beijing, China

**Keywords:** *Scutellaria baicalensis*, biosynthesis of JAs, baicalein, wogonin, 13-LOX

## Abstract

Although baicalein and wogonin contents in *Scutellaria baicalensis*, a traditional Chinese herb, are known to be regulated by jasmonic acid, the exact mechanism by which jasmonic acid regulates the accumulation of baicalein and wogonin remains unclear. In this study, we discovered SbLOX3, a gene encoding 13-lipoxygenase from the roots of *S. baicalensis*, which plays an important role in the biosynthesis of jasmonic acid. The contents of methyl jasmonate, baicalin, wogonin, and three metabolic intermediates of methyl jasmonate, 13-HPOT, OPDA, and OPC-8, were downregulated in the hair roots of the SbLOX3 RNAi lines. We confirmed that SbLOX3 was induced by drought stress simulated by PEG and *Fusarium oxysporum*, which subsequently led to changes in the content of MeJA, baicalin, and wogonin. Taken together, our results indicate that a 13-LOX is involved in the biosynthesis of jasmonic acid, and regulates the accumulation of baicalein and wogonin in *S. baicalensis* roots.

## Introduction

*Scutellaria baicalensis* Georgi is a commonly used traditional Chinese herb known for its antiviral, anti-inflammatory, and anticancer properties (Duan et al., 2014; Liu et al., 2021; Song et al., 2021). The primary medicinal metabolites found in *S. baicalensis* are baicalein and wogonin (Himeji et al., 2007; Nayak et al., 2014; Wang et al., 2018). Baicalin content is the primary evaluation index for the quality control of *S. baicalensis*, as specified in the National Pharmacopoeia of 2015 (National Pharmacopoeia Committee, 2015).

The accumulation of baicalin and wogonin in the roots of *S. baicalensis* is closely linked to a distinct anatomical structure referred to as the “root hollow”. The levels of baicalin and wogonin are observed to be higher in hollow roots as compared to those without a hollow (Ren et al., 2009; Zhao et al., 2017). This difference is related to the content of jasmonic acids (JAs) in the roots based on our previous study (Geng et al., 2023). The regulation of 4’-deoxyflavone accumulation is mediated by methyl jasmonate (MeJA), with exogenous application of MeJA leading to an increase in the levels of both baicalin and wogonin in *S. baicalensis* roots. However, the mechanism by which JAs are stimulated during root hollow development remains unknown.

The JA biosynthetic pathway and catalyzing enzymes have been elucidated in *Arabidopsis*. JA biosynthesis begins with α-linolenic acid (α-LeA) in the plastid. 13-Lipoxygenase (13-LOX) oxidizes LeA to (13*S*)-hydroperoxy octadecatrienoic acid (13-HPOT), and 13-allene oxide synthase (13-AOS) and allene oxide cyclase (AOC) catalyze 13-HPOT into 12-oxo-10,15(Z)-phytodienoic acid (OPDA; Wasternack & Hause, 2013). In *Arabidopsis*, AtLOX2, AtLOX3, AtLOX4, and AtLOX6 are four major 13-LOXs that participate in the biosynthesis of JAs. Each of these 13-LOXs participates in biosynthesis of JAs in leaves (Chauvin et al., 2013; Grebner et al., 2013; Chauvin et al., 2016). Among these genes, expression of tLOX3 and AtLOX4 is active and AtLOX2 and AtLOX6 are highly unstable (Bannenberg et al., 2009). AtLOX3 and its closely related gene, AtLOX4, are highly expressed in the roots of *Arabidopsis*, but the expression levels of AtLOX2 and AtLOX6 are very low in roots (Vellosillo et al., 2007). Thus, AtLOX3 and AtLOX4 respond to biotic and abiotic stress in roots (Ding et al., 2016; Yang et al., 2020; Oshita et al., 2023).

In our previous study, two genes, such as Sb01g66750 and Sb01g34210, were induced in roots with root hollows according to a transcriptome analysis (Geng et al., 2023). These two genes were annotated to have 13-LOX activity. In this study, we identified Sb01g66750, which was called SbLOX3, as a 13-LOX that catalyzes the biosynthesis of 13-HPOT and JAs in *S. baicalensis*. It also further affected root hollow development and the accumulation of baicalein and wogonin. We report that PEG-simulated drought stress induced the expression of SbLOX3 and the accumulation of MeJA, baicalein, and wogonin in the hair roots of *S. baicalensis*.

## Materials and methods

### Plant materials

The plants were maintained in Jinan, Shandong Province, China (36°20´N, 117°47´E). *S. baicalensis* Georgi seedlings were planted in pots (8 × 12 cm) filled with 90% nursery substrate (0–6 mm, Pindstrup, Denmark) and 10% vermiculite. The seedlings were cultured in a growth chamber at 25°C, with an illumination of 60 μmol m^−2^ s^−1^ and a humidity of 50%–75% until 10 pairs of main leaves appeared. Then, half of the seedlings were harvested, segmented by organ, frozen in liquid nitrogen, and stored at −80°C for quantitative real-time-polymerase chain reaction (qRT-PCR) analysis. The other half of the seedlings were used to induce hair roots and RNAi.

### Cloning and phylogenetic analysis of SbLOX3 and SbLOX6

The primers used for full-length cloning of Sb01g66750 and Sb01g34210 were designed from the *S. baicalensis* reference genome database of Zhao et al. (2019) and are listed in **Supplementary Table 1**. The PCR products were cloned into pDONR207 using the Gateway BP Clonase II enzyme mix (Thermo Fisher, Waltham, MA, USA). The sequencing results are shown in **Supplementary Figure 1**. The sequenced genes were aligned, and the phylogenetic trees were built using Molecular Evolutionary Genetics Analysis version 7.0 (Kumar et al., 2016) and the maximum likelihood method with 1,000 replicates of bootstrap support.

### Hair root induction for RNAi

Nonhomologous DNA regions of SbLOX3 and SbLOX6 were cloned using the primers listed in **Supplementary Table 1**. The PCR products were cloned into pDoner 207 for sequencing and then cloned into pK7WIWG2D using the Gateway LR Clonase II enzyme mix. The RNAi vector was introduced into *A. rhigogenes* A4 by electroporation. The transformants were screened on LB solid medium containing 50 mg L^−1^ spectinomycin.

The signal clone of *A. rhigogenes* A4 carrying the empty vectors or the SbLOX3 RNAi vectors was inoculated into 5 ml of YEB liquid medium containing 50 mg L^−1^ spectinomycin at 28°C and 120 rpm until an OD_600_ value of 0.5 was reached. The culture was centrifuged at 3,000 × *g* for 15 min, the supernatant was removed, and the pellet was resuspended in MS liquid medium containing 50 μM acetosyringone.

*Scutellaria* hairy roots were induced using the method described by Zhao et al. (2016) with modifications. The fourth and fifth main leaves of *S. baicalensis* seedlings were collected as leaf explants. The leaves were treated with 75% ethanol for 30 s and with 0.1% mercuric chloride for 10 min, then washed five times in sterile water. The leaves were scratched in an *A. rhigogenes*-infection solution using a knife. The leaves were dried with sterile filter paper and cultured on MS solid medium containing 50 μM acetosyringone at 25°C for 72 h in the dark. Then, the leaves were transferred to B5 medium containing 50 mg L^−1^ kanamycin for 4 weeks until the hair roots were visible. The medium was exchanged every week. The hair roots were screened by qRT-PCR.

### PEG-simulated drought stress

Hair roots with positive results were transferred to new B6 liquid medium containing 50 mg L^−1^ kanamycin with shaking at 100 rpm for 8 weeks. The liquid medium was exchanged every 2 weeks. All hair roots were transferred to a new medium for the PEG-simulated drought stress. The hair roots were randomly divided into a treatment group, in which 5%, 10%, 15%, 20%, and 25% PEG-8000 (Sigma, St. Louis, MO, USA) was added to the medium. The medium was not exchanged in the control group. The PEG-simulated drought stress lasted for 7 days. The hair roots were harvested 6 h after treatment for qRT-PCR analysis, and 1 day after treatment for MeJA content analysis and 7 days for baicalein and wogonin content. All harvested roots were frozen in liquid nitrogen and stored at −80°C for further analysis.

### Preparation of *F. oxysporum* spore suspension and inoculation on hair root

*F. oxysporum* was purchased from Agricultural Culture Collection of China. A 5-mm spore cake was cut from the edge of potato dextrose agar medium and grown in potato dextrose water media at 26°C with shaking at 200 r/min for 2 days to prepare spore suspension. Each bottle of hair roots in treatment groups was added with 2 ml of spore suspension, while the control group was added with 2 ml of potato dextrose water media. The hair roots were harvested 9 h after inoculation for qPCR analysis; 1 day after inoculation for MeJA content analysis; and 1 day, 2 days, 3 days, and 4 days after inoculation for baicalein and wogonin content analysis. All harvested roots were frozen in liquid nitrogen and stored at −80°C for further analysis.

### Wound treatment

Cultured hair roots with positive qRT-PCR results were selected for the wound treatment. The hair roots were randomly divided into a treatment group, in which the hair roots were wounded with forceps, and control plants, which were handled identically but not wounded. All hair roots were cultured for 7 days. Then, all hair roots were harvested, frozen in liquid nitrogen, and stored at −80°C for further analysis

### qRT-PCR analysis

**Figure 1:**
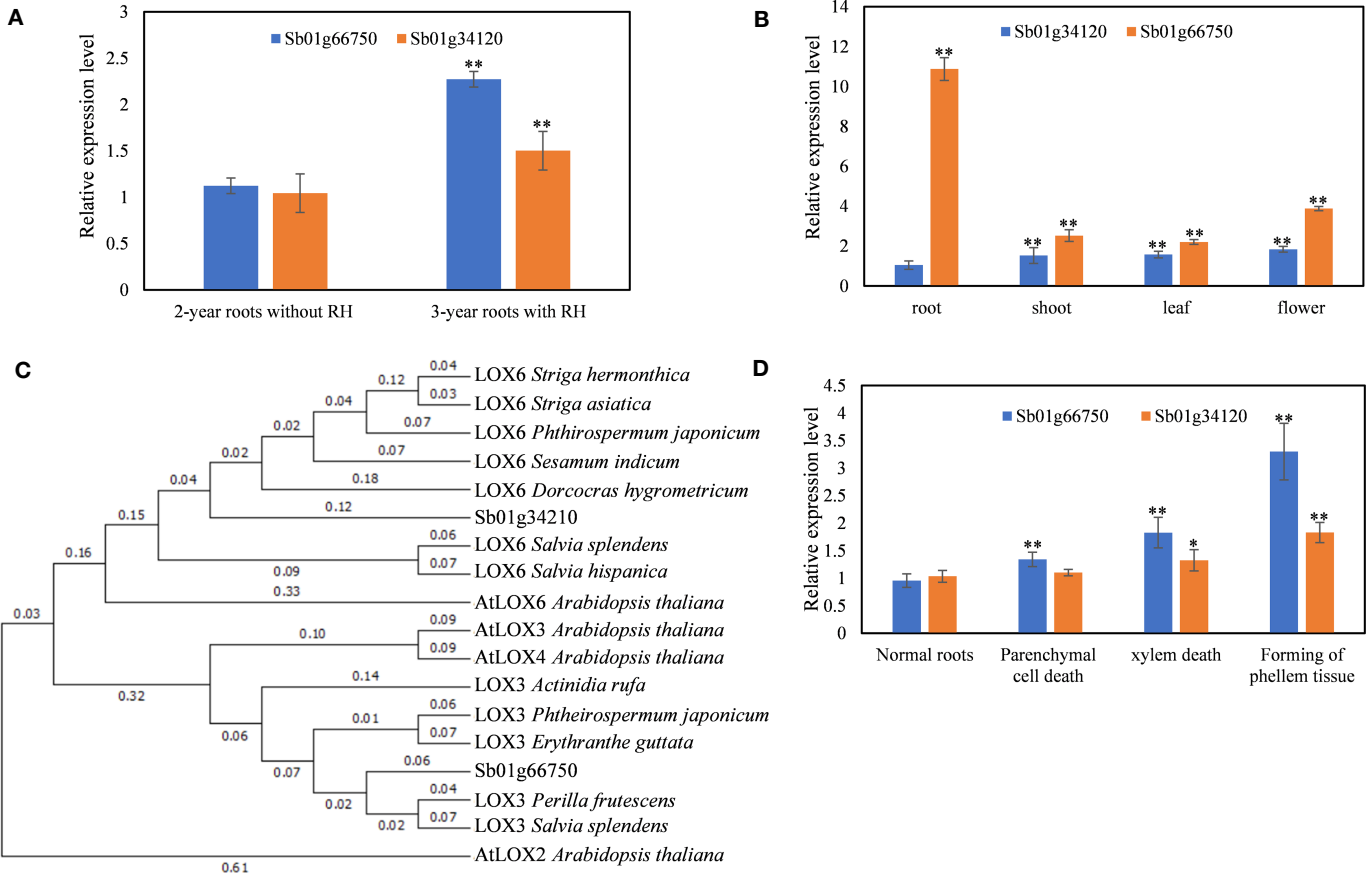
Identification and expression profiles of SbLOX genes. **(A)** Relative expression level of Sb01g66750 and Sb01g34210 in 2-year and 3-year roots with root hollow. **(B)** Relative expression level of Sb01g66750 and Sb01g34210 in different organs. **(C)** Phylogenetic tree of Sb01g66750 and Sb01g34210. **(D)** Relative expression level of Sb01g66750 and Sb01g34210 during root hollow development. The data are the means ± SDs (*n* = 3), *means *p* < 0.05, **means *p* < 0.01.

**Figure 2:**
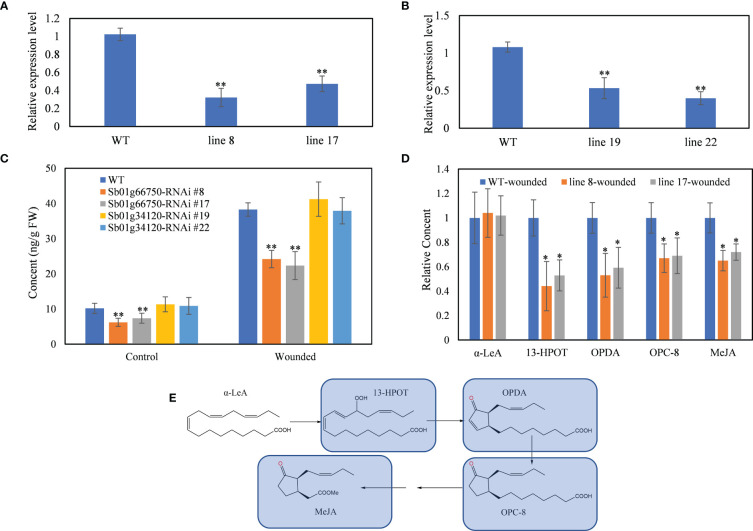
RNAi of SbLOX3 and SbLOX6. **(A, B)** Relative expression level of Sb01g66750 **(A)** and Sb01g34120 **(B)** in Sb01g66750 **(A)** and Sb01g34120 **(B)** RNAi lines. **(C)** Content of MeJA in Sb01g66750 Sb01g34120 RNAi lines with or without manual wounded. **(D)** Relative content of α-LeA, 13-HPOT, and OPDA in Sb01g66750 RNAi lines. **(E)** Biosynthesis pathway of MeJA in plants. Blue marks means downregulated compared with wild type. The data are the means ± SDs (*n* = 3), *means *p* < 0.05, **means *p* < 0.01.

**Figure 3:**
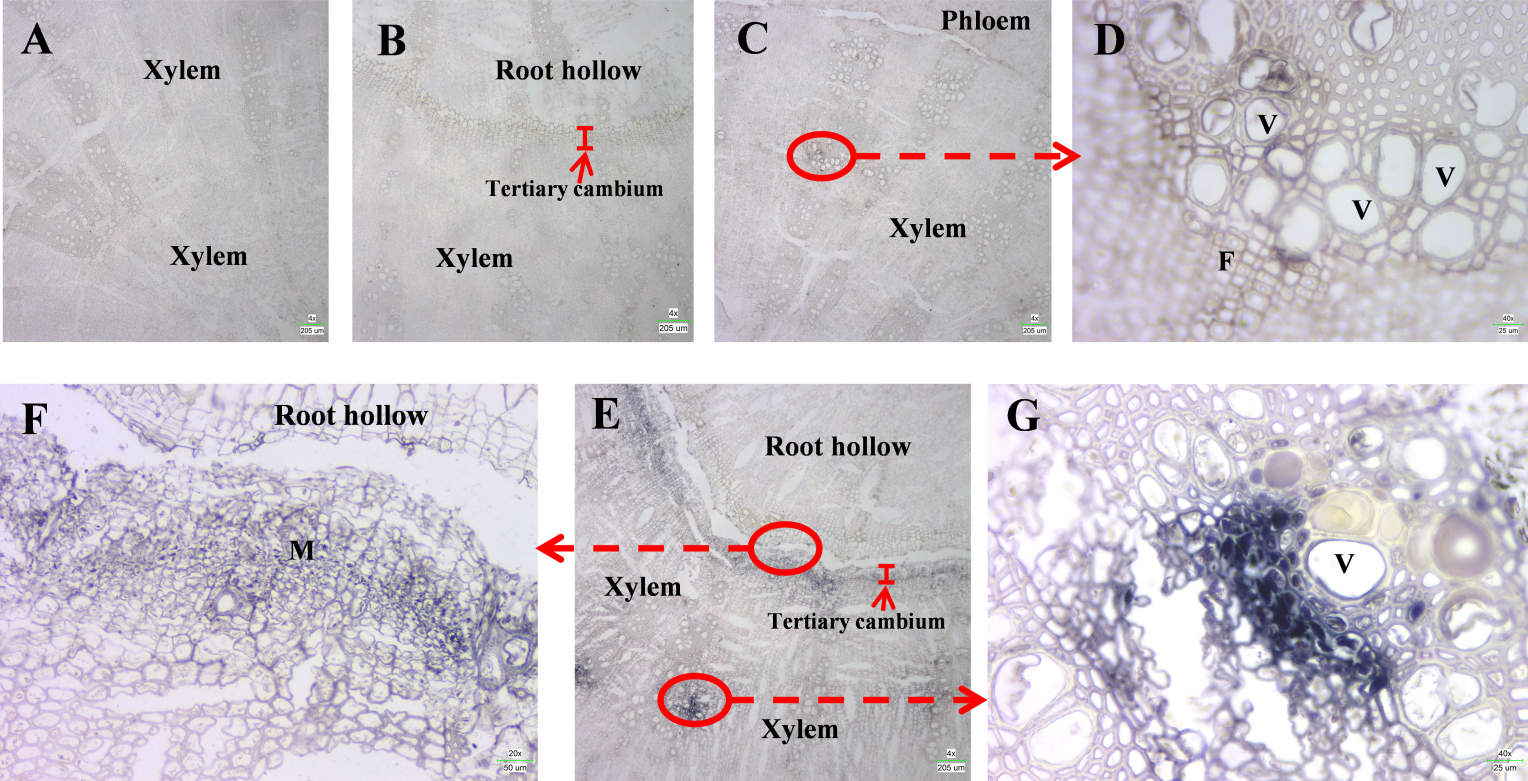
*In situ* hybridization of SbLOX3. **(A, B)** *In situ* hybridization of SbLOX3 transcripts using sense probe in root without root hollow **(A)** or roots with root hollow **(B)**. **(C)** *In situ* hybridization of SbLOX3 transcripts using anti-sense probe in root without root hollow. **(D)** Enlarged image of **(C). (E)** *In situ* hybridization of SbLOX3 transcripts using anti-sense probe in root with root hollow. **(F, G)** Enlarged image of **(E)** V, vessels. F, Fiber cells. M, meristematic cells. Scale bars are shown in the lower right corner.

The qRT-PCR analysis was performed using the method described by Geng et al. (2023) with the plant materials described by Geng et al. (2023) (**Figures 1A, C**), the frozen organ materials described above (**Figure 1B**), or with hair roots (**Figures 2**, **3**).

### UPLC-MS analysis

Baicalin, wogonin, 13-HPOT, OPDA, OPC-8, and MeJA standards were purchased from Desite Biotech (Chengdu, Sichuan Province, China). Standard stock solutions (0.1 mg ml^−1^) were prepared following Geng et al. (2023).

The frozen samples were crushed in a mixer mill (MM 400, Retsch) containing small steel balls in liquid nitrogen for 1.5 min at 30 Hz. Then, 1 g of sample was extracted three times with 10 ml of 50% methanol in an ultrasonicator bath for 30 min and centrifuged at 12,000 × *g* for 10 min to remove debris. The supernatant was collected and concentrated with a vacuum concentrator (Labconco, Kansas City, KS, USA) at 4°C until it was 2 ml. The concentrated supernatant was filtered through a 0.2-μm filter before injection. UPLC was performed with the Acquity H system (Waters Corp., Milford, MA, USA). MS was performed with the Xevo TQ-XS system (Waters Corp). Separation was achieved with a 150 × 2.1-mm, 3-µm C18-120 column (Shimadzu, Tokyo, Japan) and the following gradient: 0.1% formic acid in water (A) vs. 0.1% formic acid in acetonitrile (B) run at 0.3 ml min^−1^ and a column temperature of 40°C (0 min, 95% B; 15 min, 95% B; 16 min, 95% B; 17 min, 5% B; 20 min, 5% B).

### Statistics

All data are presented as mean ± SD. The paired or unpaired two-tailed Student’s *t-*test was used to detect group differences. *p*-values < 0.05 were considered significant. Three biological repeats were used for all analyses.

## Results

### Identification and expression profiles of the SbLOX genes

Sb01g66750 and Sb01g34120 were upregulated differentially expressed genes (DEGs) in 3-year roots with hollows compared with 2-year roots without hollows (Geng et al., 2023). To confirm the RNA-seq results, qRT-PCR was performed on 2-year roots without hollows and on 3-year roots with hollows. The results showed that Sb01g66750 and Sb01g34120 were upregulated in 3-year roots with hollows compared with 2-year roots without hollows (**Figure 1A**).

The full-length Sb01g66750 and Sb01g34120 were sequenced (**Supplemental Data 1**). A maximum-likelihood phylogenetic tree was constructed to identify the functions and the evolutionary relationships of Sb01g66750 and Sb01g34120. The results showed that Sb01g66750 was homologous with AtLOX3 and Sb01g34120 was homologous with AtLOX6 (**Figure 1B**). Thus, we named Sb01g66750 SbLOX3 and Sb01g34120 SbLOX6.

The SbLOX3 and SbLOX6 expression levels were analyzed in different organs by qRT-PCR (**Figure 1C**). The expression level of SbLOX3 was much higher than that of SbLOX6 in all organs. The expression of SbLOX3 was higher in roots than in aerial parts, and the expression of SbLOX6 was higher in aerial parts, particularly in flowers, than in roots. These organ expression patterns were consistent with those of AtLOX3 and AtLOX6 (Vellosillo et al., 2007).

MeJA content increases during the development of root hollows in *S. baicalensis* roots (Geng et al., 2023). AtLOX3 and AtLOX6 are two 13-LOX genes related to the biosynthesis of JAs (Chauvin et al., 2013; Grebner et al., 2013; Chauvin et al., 2016). Thus, qRT-PCR was used to analyze the expression levels of SbLOX3 and SbLOX6 during root hollow development (**Figure 1D**). The results showed that the expression of SbLOX3 and SbLOX6 increased 7.5-fold and 1.76-fold, respectively, during root hollow development.

### RNAi silencing of SbLOX3 and SbLOX6 in hairy roots of *S. baicalensis*

RNAi was performed to confirm the role of SbLOX3 and SbLOX6 in the biosynthesis of JAs in *S. baicalensis* roots. qRT-PCR confirmed that the expression of SbLOX3 and SbLOX6 was downregulated in the RNAi lines (**Figures 2A, B**). The SbLOX3 transcript level decreased by 78% in line 8 and by 53% in line 17. The SbLOX6 transcript level decreased by 47% in line 19 and by 60% in line 22.

MeJA content decreased from 10.17 ng/g FW to 6.19 ng/g FW in the SbLOX3 RNAi line 8 and to 7.37 ng/g FW in the SbLOX3 RNAi line 19 (**Figure 2C**). MeJA content increased significantly in the wild type (WT) and SbLOX3 RNAi lines after being wounded, but MeJA content remained significantly lower in the SbLOX3 RNAi lines than the WT. The MeJA content of the SbLOX6 RNAi lines was not significantly different from the WT before and after being wounded. Thus, we chose SbLOX3 as the major 13-LOX in the biosynthesis of JAs in *S. baicalensis* roots.

AtLOX3 in *Arabidopsis* encodes a lipoxygenase that catalyzes the oxygenation of linolenic acid, which is transformed into 13-HPOT. Thus, contents of linolenic acid, 13-HPOT OPDA, and 3-oxo-2-(20-[Z]-pentenyl)-cyclopentane-1-octanoic acid (OPC-8), which are downstream metabolites of 13-HPOT, were analyzed in the WT and SbLOX3 RNAi lines by UPLC-MS (**Figures 2D, E**). The results revealed no significant difference in linolenic acid content between the WT and SbLOX3 RNAi lines. The 13-HPOT, OPDA, and OPC-8 contents decreased significantly in the SbLOX3 RNAi lines compared with the WT. This result indicates that SbLOX3 has 13-LOX activity and catalyzes the biosynthesis of 13-HPOT and JAs in *S. baicalensis*.

The expression of JA-related transcription factors was downregulated in the SbLOX3 RNAi lines. Based on our RNA-seq results (Geng et al., 2023), we identified Sb06g11810, Sb01g24050, Sb08g14740, and Sb03g14740 as differentially expressed transcription factors associated with JAs. Among these, Sb06g11810 and Sb01g24050 were found to be homologs of AtMYC2 (**Supplemental Figure 1A**), and Sb06g11810 was specifically identified as a homolog of SmMYC2 in *Salvia miltiorrhiza*. *S. miltiorrhiza* and *S. baicalensis* belong to the Lamiaceae. Sb08g14740 was identified as a homolog of SiMYC2 in *Sesamum indicum* and Sb03g14740 was characterized as a homolog of MTB3-like in *Saliva hispanica*. We quantified the expression levels of Sb06g11810, Sb01g24050, Sb08g14740, and Sb03g29260 in both wild-type and RNAi lines using qRT-PCR. The expression of Sb06g11810 and Sb01g24050 was significantly decreased in the SbLOX3 RNAi lines, while no significant differences were observed for Sb08g14740 and Sb03g29260 (**Supplemental Figures 1B–E**).

### *In situ* hybridization confirms that SbLOX3 is expressed during the development of root hollows in *S. baicalensis*

**Figure 4:**
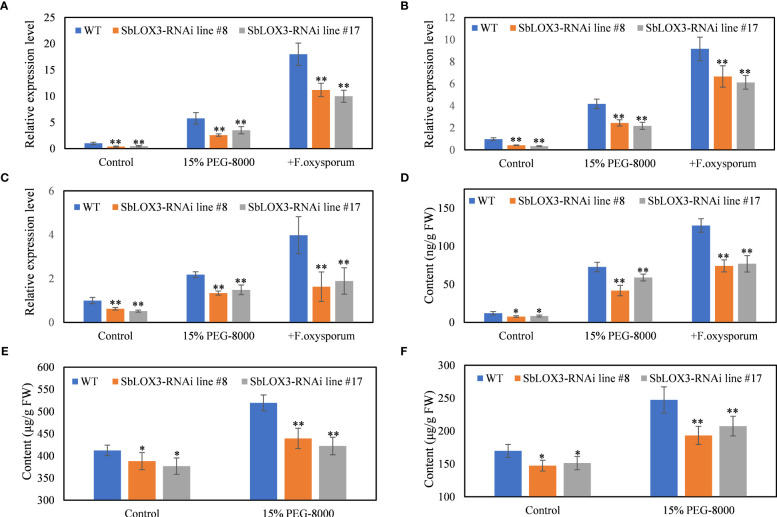
PEG-simulated drought stress on SbLOX3 RNAi lines. **(A–C)** Relative expression level of SbLOX3 **(A)**, Sb06g11810 **(B)**, and Sb01g20450 **(C)** under control and PEG-treated groups. **(D–F)** Content of MeJA **(D)**, baicalein **(E)**, and wogonin **(F)** under control and PEG-treated groups. The data are the means ± SDs (*n* = 3), *means *p* < 0.05, **means *p* < 0.01.

To confirm the function of SbLOX3 in the biosynthesis of JAs and the development of root hollows in *S. baicalensis*, DIG-labeled *in situ* hybridization was performed on roots during root hollow developmental stages I and IV (Geng et al., 2023). Only the background was detectable when using the sense probe (**Figures 4A, B**). However, strong signals were detected in tertiary cambium that wraps the root hollow in stage 4 roots and xylem in stage 1 and stage 4 roots when using the anti-sense probe (**Figures 4C, D**). The enlarged image shows that the SbLOX3 transcripts were expressed in meristem cells of the tertiary cambium (**Figure 4E**) and in the vessels and fiber cells of the xylem (**Figures 4F, G**).

### PEG-simulated drought stress and *Fusarium oxysporum* demonstrated that SbLOX3 responded to drought stress and regulated the biosynthesis of MeJA under drought stress

MeJA and other JAs respond to drought stress in plants (Wasternack and Song, 2017) and regulate the biosynthesis of baicalein in the roots of *S. baicalensis* (Xu et al., 2010; Geng et al., 2023). Thus, we hypothesized the SbLOX3 response to drought stress. Different PEG-8000 concentrations were tested to discover the optimum concentration. The results showed that the SbLOX3 expression level was higher under 15% PEG-8000 than the other PEG-8000 concentrations. qRT-PCR analysis showed that SbLOX3 was significantly induced by PEG-simulated drought stress (**Supplemental Figure 2**). The expression level of SbLOX3, Sb06g11810, and Sb01g24050 was significantly lower in the RNAi lines than in the WT under drought stress (**Figures 3A-C**). MeJA content in the hair roots of the WT and RNAi lines was induced under drought stress, and MeJA content was lower in the RNAi lines than the WT of the control and treatment groups (**Figure 3D**). Drought stress induced the biosynthesis of baicalein and wogonin in the hair roots of the WT and RNAi lines, but lower contents of baicalein and wogonin were observed in the hair roots of the RNAi lines compared with the WT (**Figures 3E, F**).

SbMYB3, an R2R3-MYB transcription factor that was reported as a regulator of biosynthesis of root-specific flavones like baicalein and wogonin in *S*. *baicalensis* roots, was induced by JAs (Fang et al., 2022). The expression level of SbMYB3 in WT and SbLOX3 RNAi lines under control and PEG-simulated drought stress and *F. oxysporum* was analyzed to confirm the role of SbLOX3 under drought stress. Results show that SbMYB3 was downregulated in SBLOX3 RNAi lines and induced by PEG-simulated drought stress (**Supplemental Figure 3**).

A noticeable trend was observed in the expression levels of SbLOX3 and MeJA content during both drought treatment and *F. oxysporum* infection. *F. oxysporum* infection resulted in the upregulation of SbLOX3, Sb06g11810, Sb01g24050, and MeJA content (**Figures 3A-D**), while the accumulation of baicalein and wogonin was significantly suppressed (**Supplemental Figure 4**). Following inoculation with *F. oxysporum*, the expression level of the reference gene β-tubulin rapidly decreased, and hair roots died at 3 days post-inoculation (**Supplemental Figures 4C, D**)

## Discussion

The roots of *S. baicalensis* contain high levels of bioactive baicalein and wogonin, and the synthesis of baicalein and wogonin in roots is regulated by JAs during the development of the root hollow (Geng et al., 2023). In this study, we established how JAs are regulated during the development of the root hollow.

### SbLOX3 was identified as the 13-LOX involved in the biosynthesis of JAs

Based on our RNA-seq results (Geng et al., 2023), SbLOX3 and SbLOX6 were DEGs related to the development of the root hollow, and this result was confirmed by qRT-PCR (**Figure 1A**). The functional annotation and phylogenetic analyses suggested that SbLOX3 and SbLOX6 have 13-LOX functions (**Figure 1D**), where SbLOX3 was homologous with AtLOX3 and SbLOX6 was homologous with AtLOX6. AtLOX3 and AtLOX6 are two 13-LOXs that participate in the biosynthesis of JAs in *Arabidopsis thaliana*. The expression level of AtLOX3 in *A. thaliana* roots is higher than that in aboveground parts. AtLOX3 regulates the growth-restricted wound response (Yang et al., 2020) and the responses to salt, heavy metal, and biotic stress (Ding et al., 2016; Chávez-Martínez et al., 2020; Oshita et al., 2023).

The expression level of SbLOX3 was significantly higher than that of SbLOX6, and the expression of SbLOX3 in roots was significantly higher than that in aerial parts (**Figure 1C**). The accumulation of MeJA, baicalein, and wogonin was suppressed in the SbLOX3 RNAi lines, but not in the SbLOX6 RNAi lines (**Figures 2C**, **3D–F**). This result indicates that SbLOX3 is involved in the biosynthesis of JAs. The RNAi and expression level results indicate that SbLOX6 may not participate in the biosynthesis of JAs or the accumulation of baicalein and wogonin.

AtLOX3 catalyzes the oxygenation of linolenic acid during the transformation into 13-HPOT in *A. thaliana* (Wasternack & Hause, 2013). No significant difference in linolenic acid content was observed between the WT and SbLOX3 RNAi lines, but 13-HPOT, OPDA, and OPC-8 content decreased significantly in the RNAi lines, indicating that SbLOX3 has 13-LOX activity in S. *baicalensis.*

### SbLOX3 is induced in the tertiary meristem during the development of the root hollow

The *in situ* hybridization results show the SbLOX3 expression pattern during the development of the root hollow (**Figure 4**). In normal roots, SbLOX3 was only expressed in vessels and xylem fibers (**Figures 4C, D**). JAs regulate the development of the xylem and the vessels (Jang et al., 2019), which was consistent with our results. The expression of SbLOX3 was significantly induced in vessels and xylem fibers of stage IV roots during root hollow development, compared with normal roots (**Figures 4E, G**). SbLOX3 was induced in tertiary cambium during root hollow development (**Figure 4F**). The JA content results during stage IV were consistent with our MeJA results (Geng et al., 2023 and **Figure 2C**).

Based on our previous study (Geng et al., 2023), JAs regulate the accumulation of baicalein and wogonin during root hollow development in *S. baicalensis.* SbLOX3 expression was induced during the development of the root hollow (**Figure 1C**), and SbLOX3 expression was related to the accumulation of MeJA *in vivo* (**Figure 2**). The location of SbLOX3 in tertiary cambium during root hollow development indicated that SbLOX3 and JAs participate in the development of the root hollow.

### SbLOX3 is induced under drought stress and *Fusarium oxysporum*

JAs respond to drought stress (Yao et al., 2022; Liu et al., 2021) and *Fusarium oxysporum* infection (Hernández-Aparicio et al., 2021; Pazarlar et al., 2022). Our results confirm the expression of SbLOX3 and that the accumulation of MeJA was induced by PEG-simulated drought stress and *F. oxysporum* in the hair roots of *S. baicalensis* (**Figures 3A, B**). The accumulation of baicalein and wogonin was induced by PEG-simulated drought (**Figures 3C, D**) but suppressed by *F. oxysporum* (**Supplemental Figures 3A, B**). The contents of MeJA, baicalein, and wogonin decreased in the SbLOX3 RNAi lines, compared with the WT (**Figures 3B-D**), indicating that drought stress induced the expression of SbLOX3 and the accumulation of MeJA, baicalein, and wogonin. *F. oxysporum* infection caused a rapid decrease in the expression level of the qRT-PCR reference geneβ-tubulin (**Supplemental Figure 3C**) and the death of hair roots (**Supplemental Figure 3D**). The suppression of baicalein and wogonin by *F. oxysporum* may be attributed to the death of the hair roots, which were unable to withstand the *F. oxysporum* infection (**Supplemental Figure 3**).

The expression level of SbMYB3 under drought stress supports our results. SbMYB3 was an R2R3-MYB transcription factor that is induced by JAs. SbMYB3 is a positive regulator for baicalein and wogonin biosynthesis by directly binding to the promoter of SbFNSII-2 and enhances its activity. Upregulated SbMYB3 induced the expression of SbFNSII-2 (Fang et al., 2022). Our results confirmed that SbMYB3 was downregulated in SbLOX3 RNAi lines and upregulated by PEG-simulated drought stress. These results explain how baicalein and wogonin contents were regulated by SbLOX3 and PEG-simulated drought stress.

### Sb06g11810 and Sb01g24050 have the potential to function as MYC2 in *S. baicalensis*

Sb06g11810 and Sb01g24050 were identified as homologs of AtMYC2, whereas Sb06g11810 was identified as a homolog of SmMYC2. These two genes have the potential to function as MYC2 orthologs in *S. baicalensis* (**Supplemental Figure 1**). MYC2 plays a pivotal role as a regulatory factor in the JA signaling pathway, responsible for coordinating plant responses to both biotic and abiotic stresses (Kazan and Manners, 2013). Liu et al. (2022) provided evidence that SmMYC2 functions as a positive regulator in the biosynthesis of phenolic acids and anthocyanins in *S. miltiorrhiza.* Notably, Sb06g11810 and Sb01g24050 were induced during root hollow development (Geng et al., 2023), PEG-simulated drought stress, and *F. oxysporum*, but their expression was suppressed in SbLOX3 RNAi lines (**Figures 3A–C**; **Supplemental Figure 1**). The specific functions of Sb06g11810 and Sb01g24050, however, warrant further investigation. While their homology with AtMYC2 and SmMYC2 suggests that they might function similarly to MYC2 in *S. baicalensis*, additional studies are necessary to confirm this hypothesis.

Taken together, we identified SbLOX3 as a 13-LOX that catalyzes the biosynthesis of 13-HPOT and JAs in *S. baicalensis* and that affected the accumulation of baicalein and wogonin. PEG-simulated drought stress induced the expression of SbLOX3 and the accumulation of MeJA, baicalein, and wogonin in the hair roots of *S. baicalensis*. Flavonoid metabolites are induced to resist drought in plants (Yang et al., 2020; Meng et al., 2021; Yang et al., 2021; Tiedge et al., 2022). Baicalein and wogonin are two major pharmacodynamic flavonoids in *S. baicalensis*. Thus, this study provides new insight into the management of *S. baicalensis* cultivation after further confirmation in the field. This study also established the identification of MYC2 in *S. baicalensis* and further investigation of the JA-MYC2 signaling pathway.

## Data availability statement

The original contributions presented in the study are included in the article/**Supplementary Material**. Further inquiries can be directed to the corresponding author.

## Author contributions

DG, XW, and LG designed the experiments. DG, WL, and RW performed the experiments. DG, YZ, and XW wrote the manuscript. DG analyzed the data. All authors contributed to the article and approved the submitted version.
