# Supplementary figures and images for: A 13-LOX participates in the biosynthesis of JAs and is related to the accumulation of baicalein and wogonin in Scutellaria baicalensis
Source: Front Plant Sci. 2023 Jul 13;14:1204616. doi: 10.3389/fpls.2023.1204616 (PMC10373884; doi:10.3389/fpls.2023.1204616)

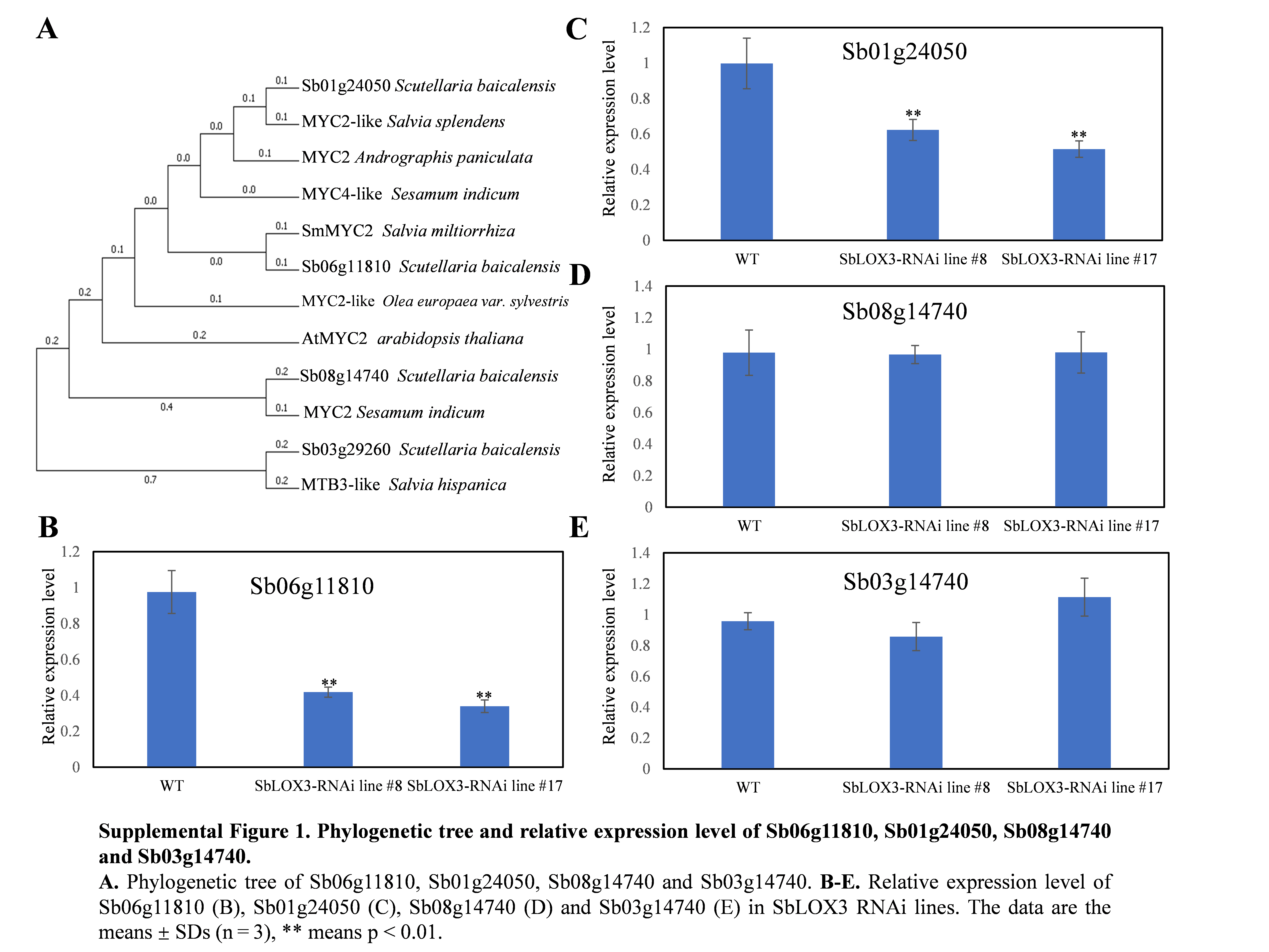

Supplement: Supplementary file 1 [file Image_1.tif]

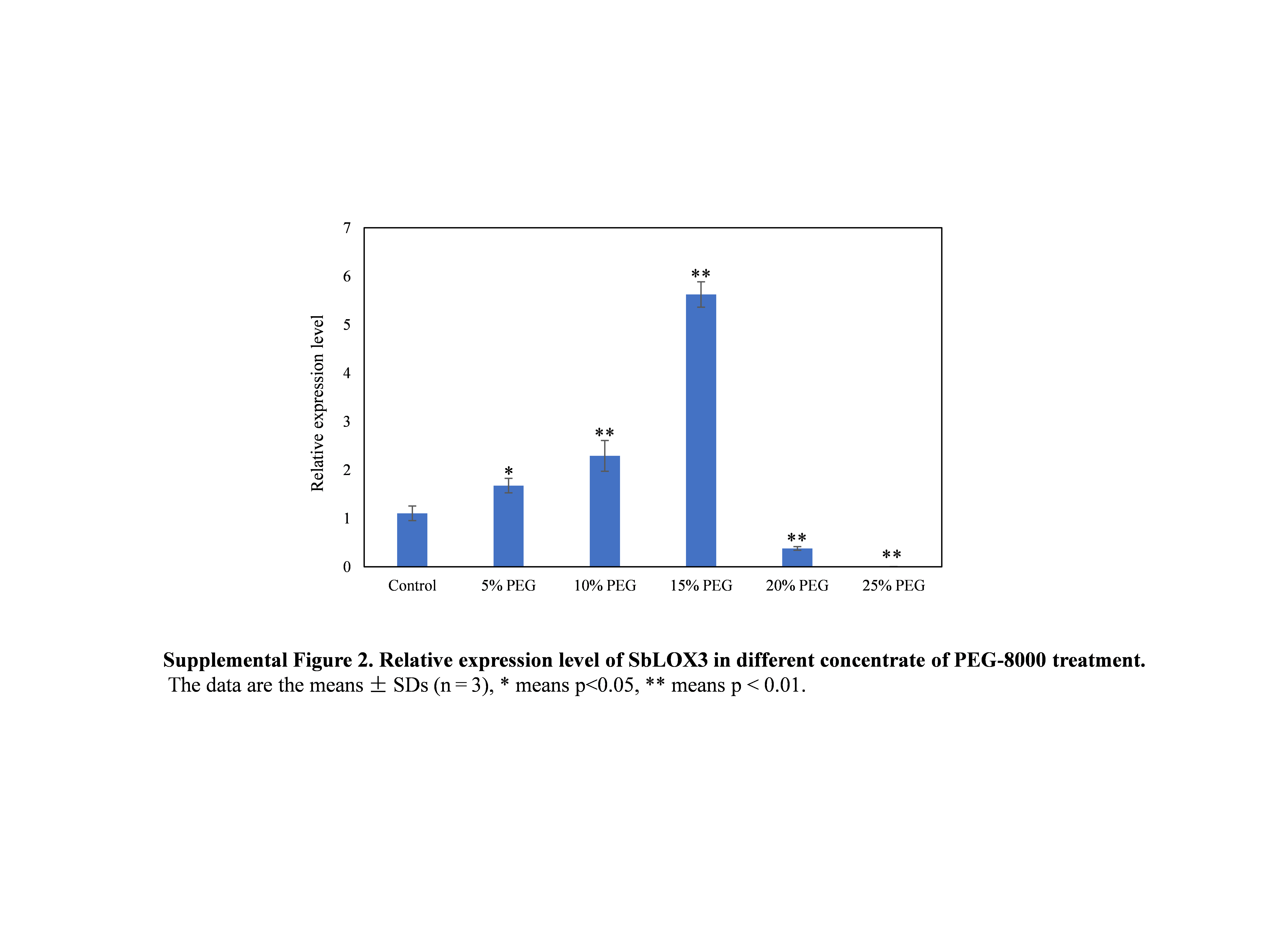

Supplement: Supplementary file 2 [file Image_2.tif]

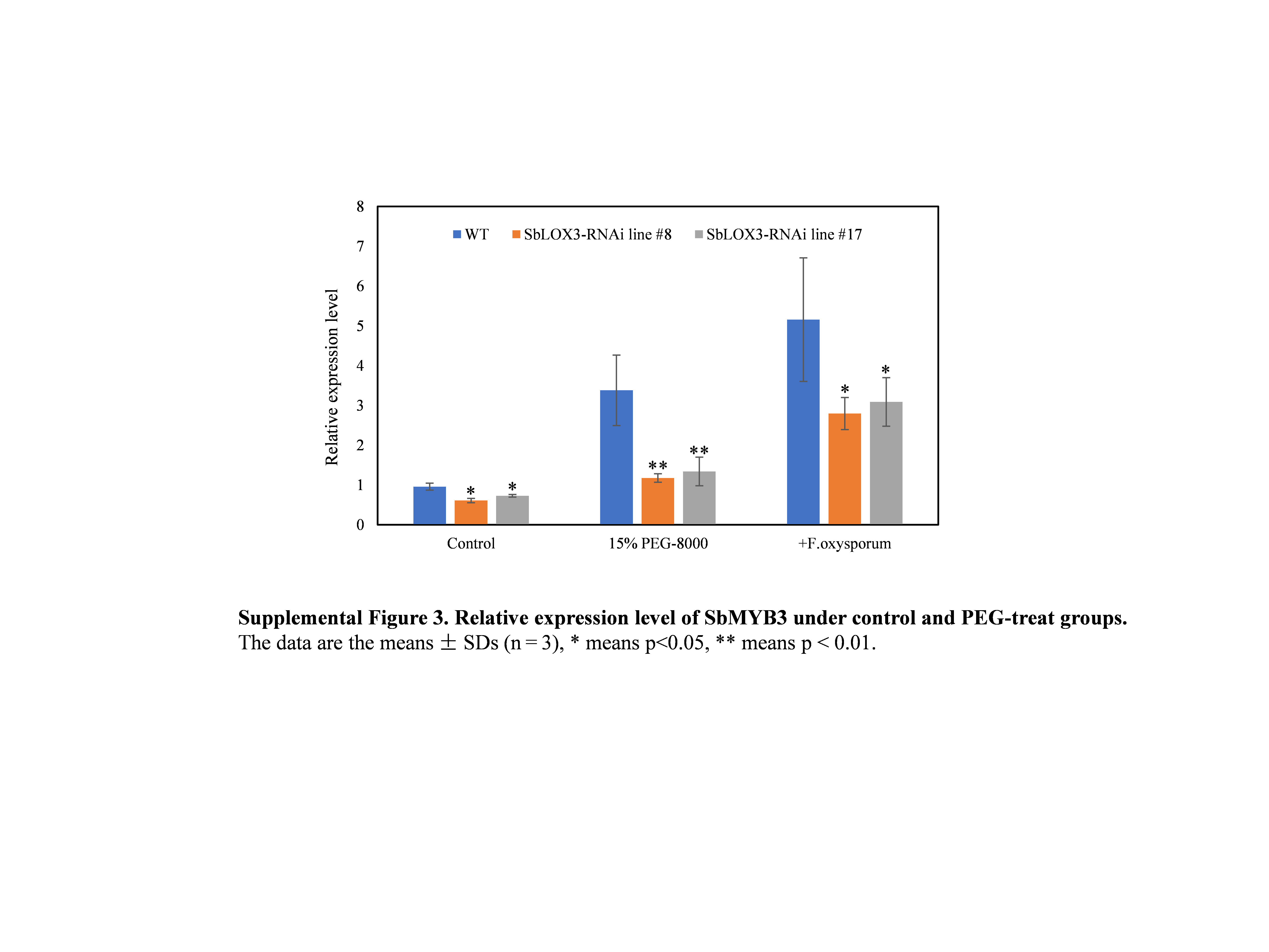

Supplement: Supplementary file 3 [file Image_3.tif]

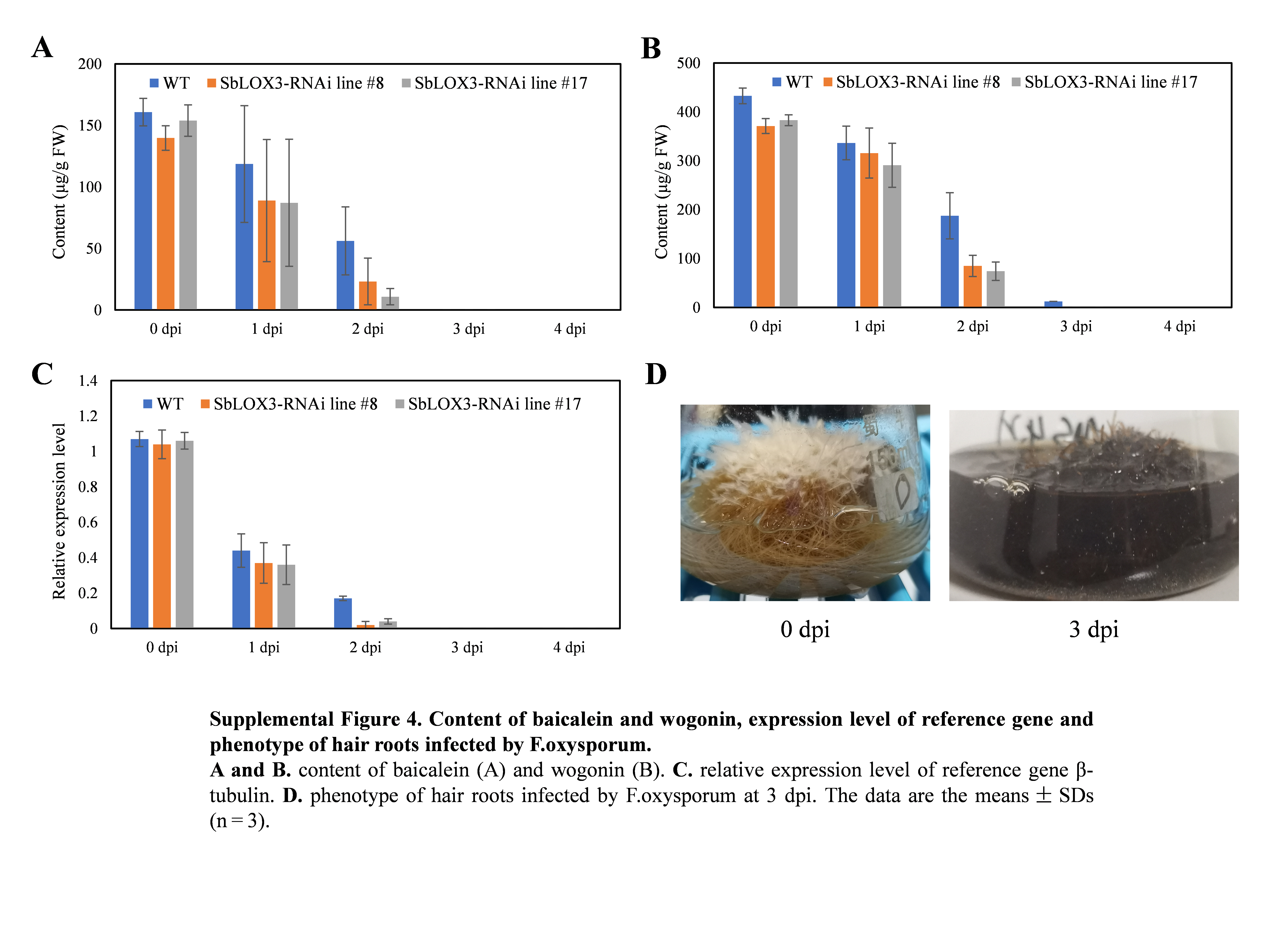

Supplement: Supplementary file 4 [file Image_4.tif]

Monday, March 20, 2023 02:57 PM

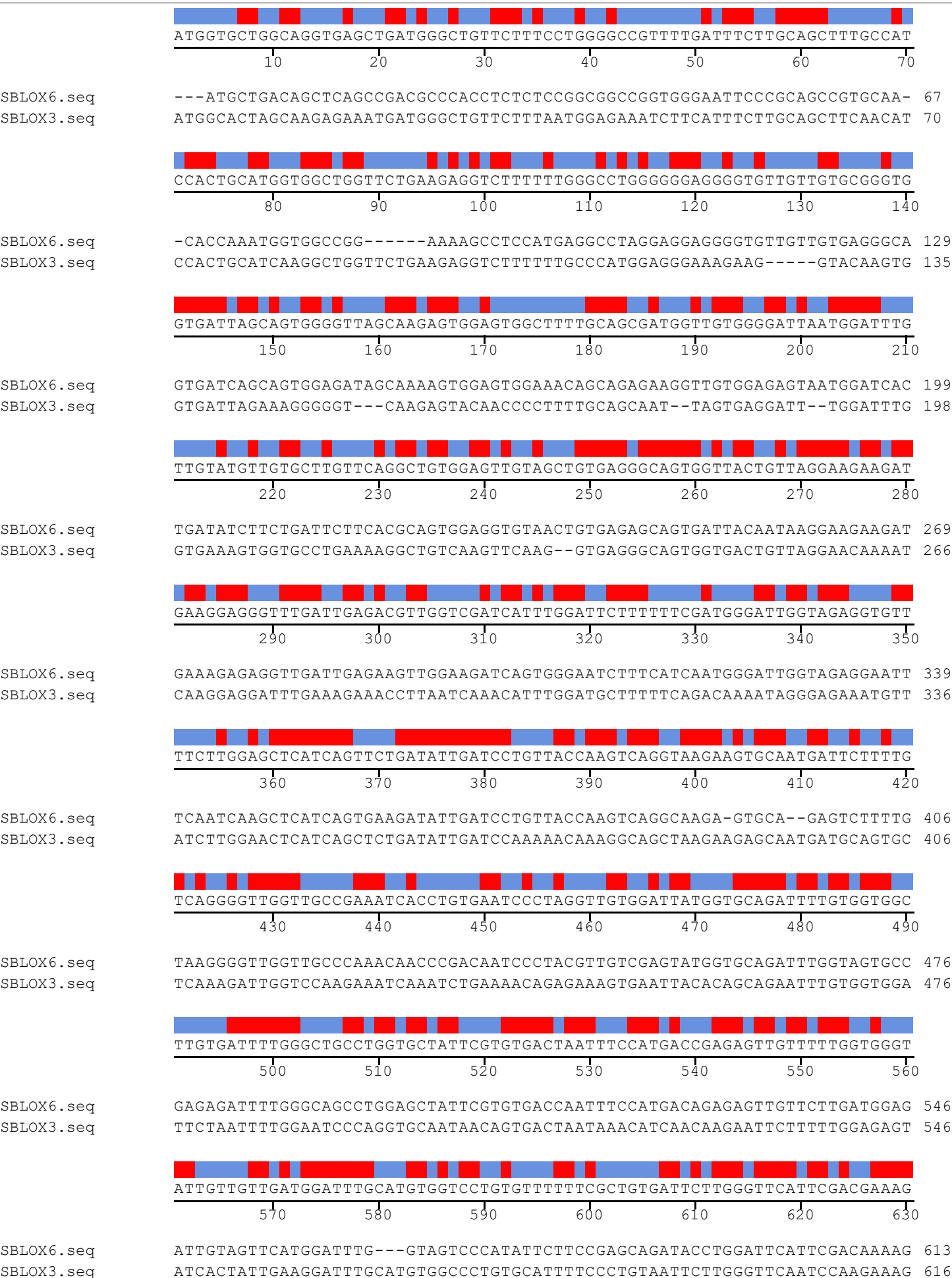

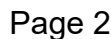

Monday, March 20, 2023 02:57 PM

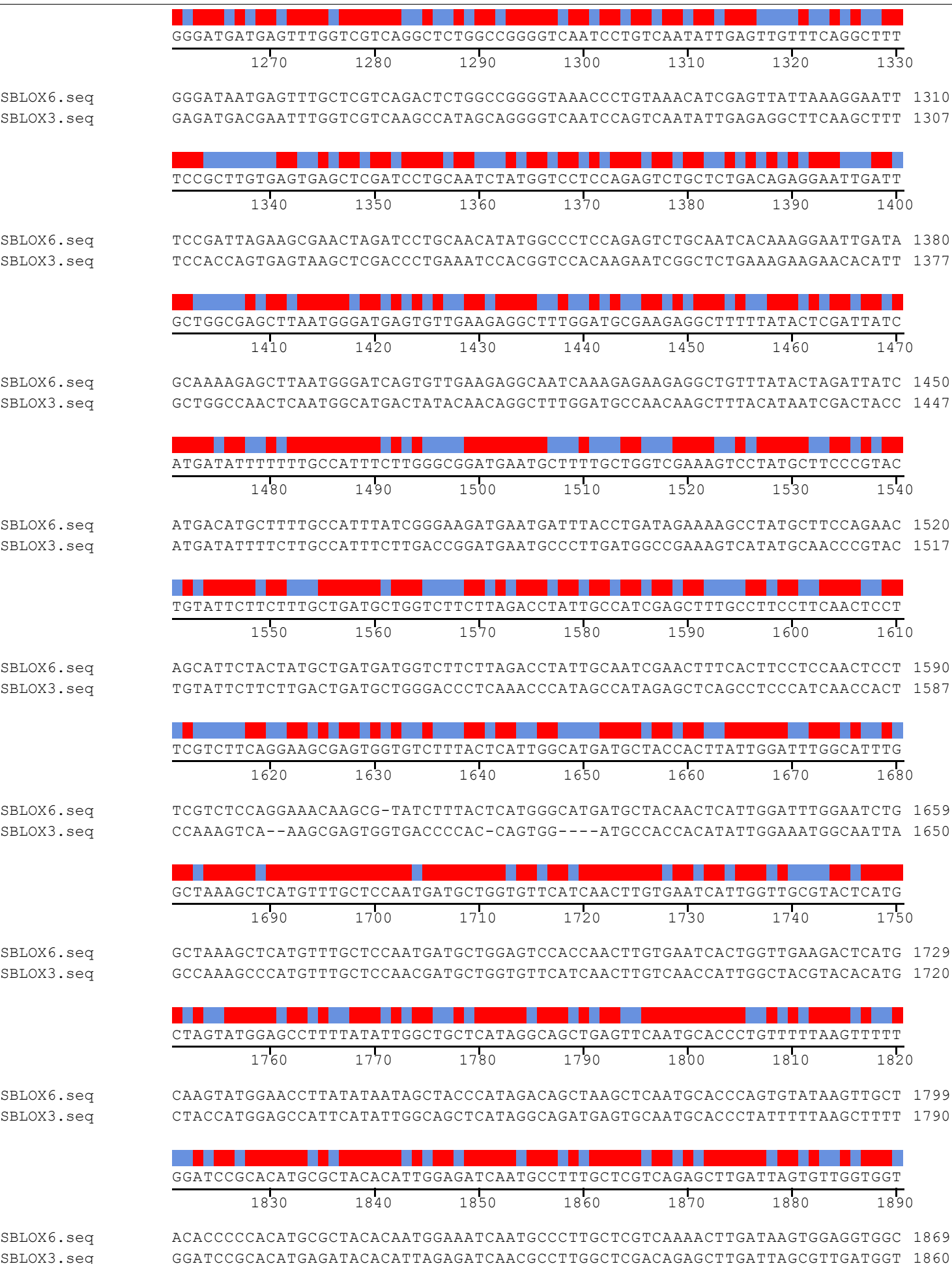

Monday, March 20, 2023 02:57 PM

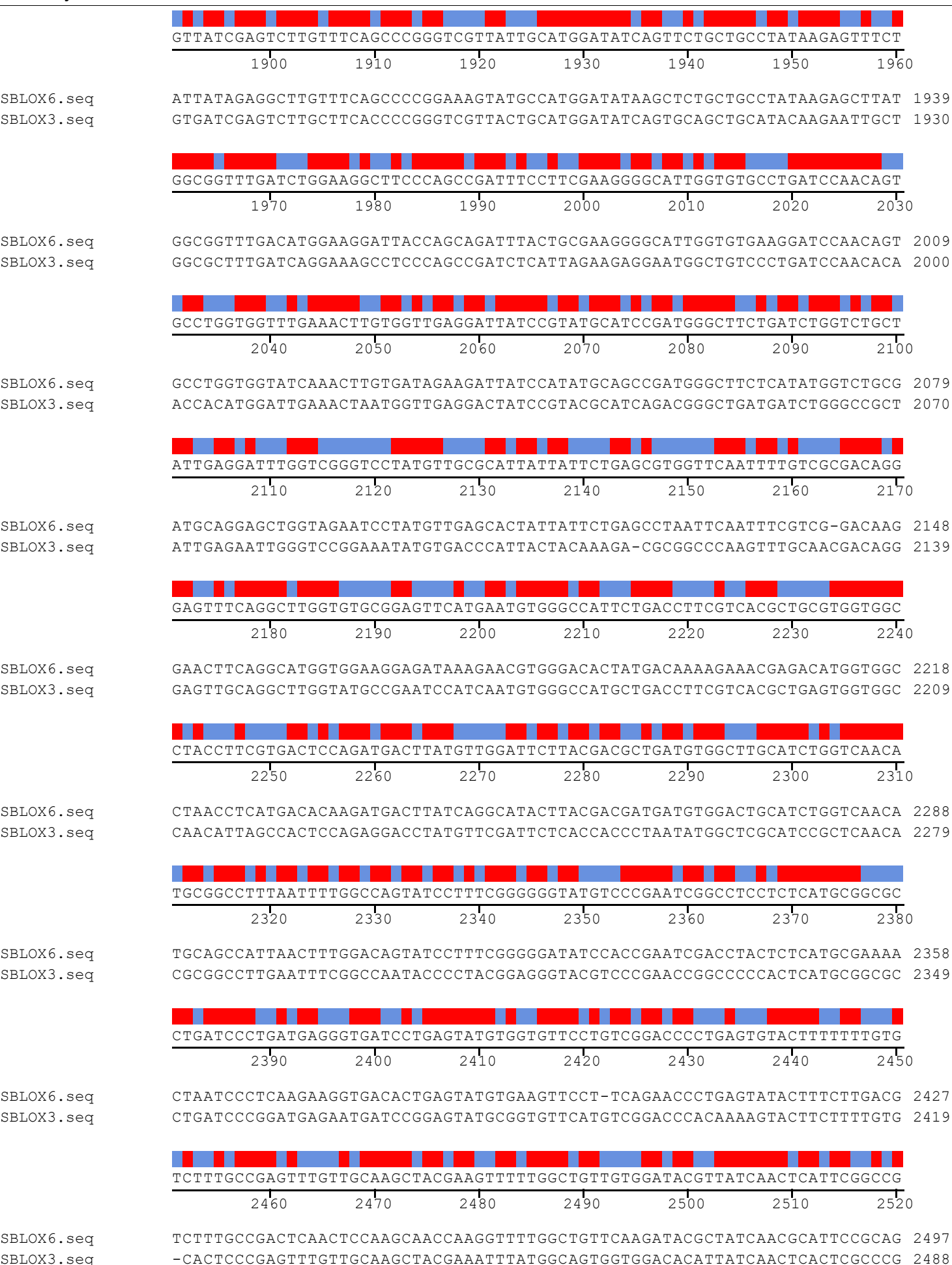

Monday, March 20, 2023 02:57 PM

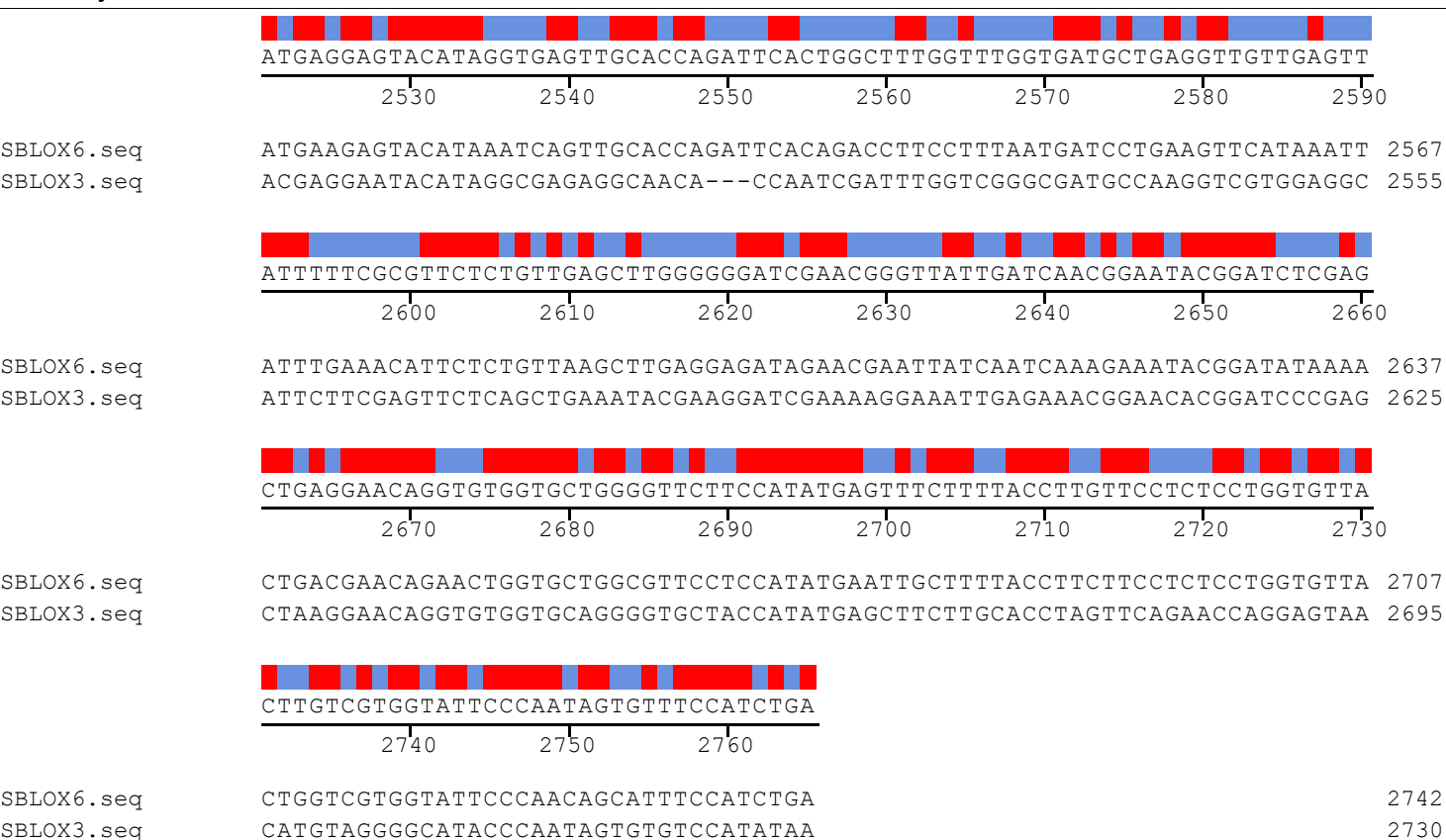

Supplement: Supplementary file 6 [file DataSheet_1.pdf]
